# Elevated *OPRD1* promoter methylation in Alzheimer’s disease patients

**DOI:** 10.1371/journal.pone.0172335

**Published:** 2017-03-02

**Authors:** Huihui Ji, Yunliang Wang, Guili Liu, Lan Chang, Zhongming Chen, Dongsheng Zhou, Xuting Xu, Wei Cui, Qingxiao Hong, Liting Jiang, Jinfeng Li, Xiaohui Zhou, Ying Li, Zhiping Guo, Qin Zha, Yanfang Niu, Qiuyan Weng, Shiwei Duan, Qinwen Wang

**Affiliations:** 1 Zhejiang Provincial Key Laboratory of Pathophysiology, School of Medicine, Ningbo University, Ningbo, Zhejiang, China; 2 Department of Neurology, the 148 Central Hospital of PLA, Zibo, Shandong, China; 3 Ningbo Kangning Hospital, Ningbo, Zhejiang, China; 4 Department of Internal Medicine for Cadres, the First Affiliated Hospital of Xinjiang Medical University, Urumchi, China; 5 Ningbo No. 1 Hospital, Ningbo, Zhejiang, China; 6 School of Medicine, Lishui University, Lishui, Zhejiang, China; 7 The Affiliated Hospital of Ningbo University, Ningbo, Zhejiang, China; Duke University, UNITED STATES

## Abstract

Aberrant DNA methylation has been observed in the patients with Alzheimer’s disease (AD), a common neurodegenerative disorder in the elderly. *OPRD1* encodes the delta opioid receptor, a member of the opioid family of G-protein-coupled receptors. In the current study, we compare the DNA methylation levels of *OPRD1* promoter CpG sites (CpG1, CpG2, and CpG3) between 51 AD cases and 63 controls using the bisulfite pyrosequencing technology. Our results show that significantly higher CpG3 methylation is found in AD cases than controls. Significant associations are found between several biochemical parameters (including HDL-C and ALP) and CpG3 methylation. Subsequent luciferase reporter gene assay shows that DNA fragment containing the three *OPRD1* promoter CpGs is able to regulate gene expression. In summary, our results suggest that *OPRD1* promoter hypermethylation is associated with the risk of AD.

## Introduction

As the most prevalent type of dementia worldwide [1], Alzheimer’s disease (AD) has become one of the most common neurodegenerative disorders among the elderly. AD patients show cognitive decline and motor function reduction, and this reduces their quality of life.

The pathogenesis of AD remains unclear. Both environmental and genetic factors have been implicated in the progression of AD [2]. Epigenetics may be a bridge between environmental and genetic factors [3]. DNA methylation, an important epigenetic mechanism, can remodel hereditary features without nucleic acid sequence changes. Moreover, changes of gene DNA methylation levels have been found to be associated with AD [4]. Previously, we have also observed significantly elevated DNA methylation levels of *BDNF* and *OPRK1* promoters in AD patients [5,6].

*OPRD1* encodes delta opioid receptor, a member of the opioid family of G-protein-coupled receptors. Delta opioid receptor is important in cognitive function and mood driven behavior [7]. A quantitative autoradiography study shows that binding of delta-opioid receptors is decreased in amygdaloid complex and ventral putamen in AD patient’s brains [8]. Other studies have found that delta opioid receptor contributes to the formation of β- and γ-secretase complexes, which play a key role in the generation of Aβ [9,10]. Aβ-related pathology and Aβ-dependent behavioral deficits are ameliorated in mice after the knockdown or antagonization of delta opioid receptor [10], suggesting that *OPRD1* may be associated with AD. In addition, early studies suggest that the amyloidogenic processing of APP is enhanced upon delta opioid receptor activation, and the selective antagonist-mediated modulation of delta opioid receptor may provide a novel treatment strategy against AD [9,10].

In light of previous findings, we recruit a set of case-control samples and test whether *ORPD1* promoter methylation is correlated with AD.

## Materials and methods

### Sample and phenotype collection

A total of 51 sporadic AD patients (age range: 53–96) and 63 normal controls (age range: 62–93) are recruited from Ningbo No.1 Hospital and Ningbo Kangning Hospital. The mean (SD) duration of AD is 9.5 (6.5) years. A series of tests are performed according to ICD-10. The suspected dementia patients have received a complete set of nervous system examinations. The cognitive function of participant is determined by the mini-mental state examination (MMSE), montreal cognitive assessment scale (MoCA), and actmty of daily living (ADL) scales. The Hachinski scale is used to exclude the vascular dementia patients. The depressive patients are excluded by the Hamilton's Depression Scale. All the AD patients without a family history are independently diagnosed by two experienced neurological physicians (CZ and ZQ). Normal controls are identified through physical and mental health examinations. All the involved individuals are Han Chinese residing in Ningbo City. The detailed information about subjects is presented in Table 1. The study protocol is approved by the Ethical Committees at Ningbo University, Ningbo No.1 Hospital, and Ningbo Kangning Hospital. Written informed consent is obtained from all subjects or from their guardians. The detection methods for the biochemical parameters are performed as described previously [5].

**Table 1:** Characteristics of AD cases and controls ^a^^,^ ^b^.

characteristics	AD cases	controls	*p* value
age (year)	80.94±8.88	79.78±7.87	0.463
male/female	27/24	46/17	0.026
BMI	22.39±3.60	22.63±3.04	0.704
hypertension	30/51	46/63	0.116
smoking	4/51	15/63	**0.025**
diabetes	15/51	23/63	0.549
TG (mmol/L)	1.33±0.74	1.40±0.97	0.688
TC (mmol/L)	4.44±1.02	4.22±1.23	0.346
HCY (μmol/L)	19.73±10.61	17.72±20.44	**0.046**
Glu (mmol/L)	5.18±1.57	5.49±2.67	0.389
TBIL (μmol/L)	8.61±2.99	14.23±6.23	**4.81E-07**
DBIL (μmol/L)	3.68±1.30	6.44±2.94	**1.06E-07**
IBIL (μmol/L)	4.95±1.85	7.78±3.69	**1.44E-05**
K (mmol/L)	4.34±0.422	3.84±0.43	**1.45E-04**
P (mmol/L)	1.25±0.15	1.12±0.28	**0.036**
HDL-C (mmol/L)	1.12±0.27	1.02±0.30	0.077
ALP (U/L)	1.88±0.12	1.94±0.17	0.135
Lp(a)	1.75±2.34	0.35±0.27	**0.005**

^a^: *p* values < 0.05 are in bold fonts. Two independent-samples t-test is used to assess the differences in the mean values of the continuous variables between the AD cases and controls. TG: triglyceride; TC: cholesterol; HCY: Homocysteine; Glu: glucose; TBIL: total bilirubin; DBIL: direct bilirubin; IBIL: indirect bilirubin; K: potassium; P: phosphorus; HDL-C: high-density lipoprotein cholesterol; ALP: alkaline phosphatase; Lp(a): lipoprotein(a).

^b^: Log-transformation is used for HCY and ALP.

### Bisulfite pyrosequencing assay

Genomic DNA extraction and quantification procedures are as described previously [11]. The sodium bisulfite converted DNA is analyzed using pyrosequencing technology (Pyromark PCR Kit, Qiagen, Dusseldorf, Germany). The reverse strand is pyrosequenced by Qiagen Pyromark Q24 machine (Qiagen, Dusseldorf, Germany). PCR primers are designed using PyroMark Assay Design software. The sequences are 5'-biotin-TTGAGAGAATGAATGGGGTGTAAGAAA-3' for the forward primer, 5'-TACCACCTCCCTCAAAAAATAATTAA-3' for the reverse primer and 5'-AATAATTAAAAAAATTAAATAAAAT-3' for the sequencing primer.

### Cell culture

Human HEK293T cells (catalogue number, *GNHu17*)are purchased from cell bank of Chinese Academy of Sciences (Shanghai, China), and they are maintained in Dulbecco’s modified Eagle’s medium (DMEM, HyClone, Logan, Utah, USA) supplemented with 10% fetal bovine serum (FBS, TransGen Biotech, Beijing, China) at 37°C in a humidified incubator with a 5% CO_2_ atmosphere.

### Construction of recombinant plasmids

A promoter fragment containing the upstream sequence from -459 to -143 bp of *OPRD1* have amplified with the forward primer (5’-ATTTCTCTATCGATAGGTACCGAATGAATGGGGTGCAAGAAAGGCACG-3’) and the reverse primer (5’-ATGCAGATCGCAGATCTCGAGGCGACCCCGTCCCAACCTGGACT-3’). A Gel Extraction Kit (Omega, Norcross, GA, USA) is used to gel purify the PCR product. The fragment is digested with XhoI and KpnI (New England Biolabs, Ipswich, England), purified by a Cycle Pure Kit (Omega, Norcross, GA, USA), then cloned into the pGL3 Basic vector using a DNA Ligation Kit (TaKaRa, Japan). All the primers are synthesized by Invitrogen company (Shanghai, China).

#### Transfection and reporter gene activity assay

HEK293T cells are cultured in 24-well plates at a density of 0.5×105/well in 500 μl DMEM with 10% FBS. Twelve hours after seeding, the medium is replaced and co-transfected with the recombinant pGL3 vector and pRL-SV40 according to the manufacturer’s protocol (TransLipid HL Transfection Reagent, TransGen Biotech, Beijing, China). The medium is replaced with fresh DMEM with 10% FBS after 4–6 hours of co-transfection. After 18–72 hours of transfection, 293T cells are harvested and lysed according to the manufacturer’s protocol (Dual-Luciferase^®^ Reporter Assay Systems, Promega, Madison, USA). Renilla and firefly luciferase activities are detected using SpectraMax 190 (Molecular Devices, California, USA). The pGL3 Promoter Vector (Promega, Madison, USA), containing an SV40 promoter upstream of the Firefly luciferase gene, is used as a positive control. Additionally, the pRL-SV40 vector (Promega, Madison, USA) containing the Renilla luciferase gene is used as an internal control in this study.

### Statistical analyses

All the statistical analyses are performed using the Statistical Program for Social Sciences (SPSS) software, version 16.0 (SPSS, Inc., Chicago, IL, USA). The two independent-samples t-test is used to assess the differences in the mean values of the continuous variables. The chi-square test is performed to assess the differences of categorical variables. The logistic regression is conducted to correct the smoking status. The Pearson or Spearman correlation test is used to detect the associations between *OPRD1* methylation and the metabolic characteristics. Power analysis is estimated with the PASS 14 Power Analysis and Sample Size Software (NCSS, Utah, USA). The means and the standard deviations of three CpG sites have shown in Table 2. The results are considered to be significant when p value is < 0.05.

**Table 2:** Comparisons of *OPRD1* methylation levels between AD cases and controls.

total	AD cases	controls	*p* value	*p* value^#^	power
CpG1 (%)	86.22±7.79	83.89±8.50	0.052	0.107	0.303
CpG2 (%)	87.78±4.59	86.37±4.93	0.094	0.174	0.325
CpG3 (%)	77.96±4.08	74.41±3.98	**1.68E-07**	**2.12E-04**	0.995

A nonparametric rank test is applied, and the bold type indicates significant differences between the AD cases and controls.

^#^: The *p* value is adjusted for gender and history of smoking.

## Results

**Fig 1:**
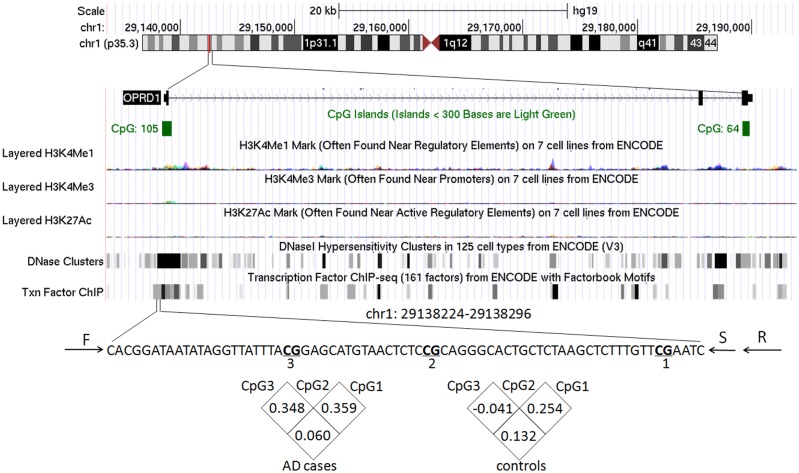
Schematic visualization of *OPRD1* and correlation of methylation levels among three CpG sites*. *: F, R, and S stand for forward, reverse, and sequencing primers, respectively.

As shown in Fig 1, there are two CpG enriched regions in *OPRD1* gene. Here, we focus on the promoter CpG island with 105 CpG dinucleotides, where are overlapped with H3K4me1 and H3K4me3 binding sites. We design pyrosequencing primers for amplification and sequencing using the PyroMark Assay Design software. We select a set of primers with the best score for the current methylation study. Our results show that the methylation levels of three CpG sites are not correlated with each other according to the coefficients among the three CpG sites. Therefore, we perform the association tests for the three CpG sites separately.

The biochemical parameters for the cases and controls are measured and analyzed in our study. Among these biochemical parameters, lipoprotein A [Lp(a)] is shown significantly higher in AD patients compared to controls (*p* = 0.005, Table 1). Homocysteine (HCY), total bilirubin (TBIL), direct bilirubin (DBIL), indirect bilirubin (IBIL), potassium (K), and phosphorus (P), show significant differences between the AD cases and controls (HCY: *p* = 0.046, TBIL: *p* = 4.81E-07, DBIL: *p* = 1.06E-07, IBIL: *p* = 1.44E-05, K: *p* = 1.45E-04, P: *p* = 0.036, Table 1).

Significantly higher CpG3 methylation levels are found in AD cases than in controls, even after adjusting for gender and history of smoking (CpG1: *p* = 0.052, corrected *p* = 0.107; CpG2: *p* = 0.094, corrected *p* = 0.174; CpG3: *p* = 1.68E-7, corrected *p* = 2.12E-4; Table 2). Our results show CpG3 has a power of 0.996, whereas CpG1 and CpG2 only have power values of 0.303 and 0.325 in the comparisons of their methylation levels between AD cases and controls. This suggests that negative association results of CpG1 and CpG2 may need to be further evaluated with a larger sample size. In addition, no difference between genders is found in the methylation levels at the three CpG sites (*p* > 0.05, data not shown).

We perform drug-based subgroup case-control comparisons for the methylation of all the three CpG sites. Our results show that CpG1 methylation remains no association with AD between the drug-treated subgroups and controls (S1 Fig). One significant association for CpG2 methylation is observed between unknown-treated AD cases and controls (*p* = 0.018). As for CpG3 methylation, nearly all the AD subgroups except memantine-treated AD cases show significantly higher methylation than controls (*p* < 0.05).

**Fig 2:**
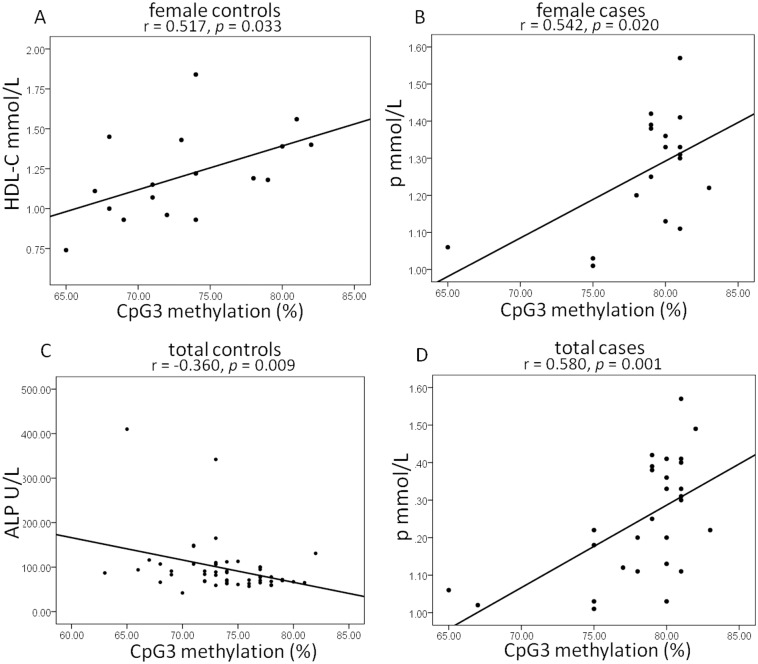
Significant correlations between the biochemical parameters and *OPRD1* promoter methylation in CpG3*. *: The number of participants with phosphorus index is 28.

Significant associations are observed between CpG3 methylation and several biochemical parameters. While in control subjects, ALP in total controls (*p* = 0.009) and HDL-C in female controls (*p* = 0.033) are significantly correlated with CpG3 methylation (Fig 2). Other metabolic indexes show no statistical correlations with CpG3 methylation. Our results show that phosphorus indexes are positively correlated with CpG3 methylation in total and female AD cases (total: *p* = 0.001, female: *p* = 0.020).

**Fig 3:**
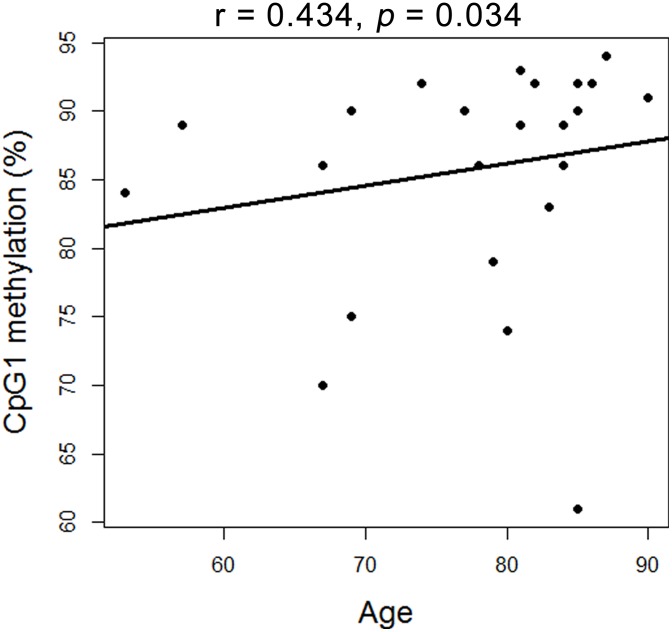
Correlation between age and methylation of *OPRD1* promoter CpG1 in female patients.

**Fig 4:**
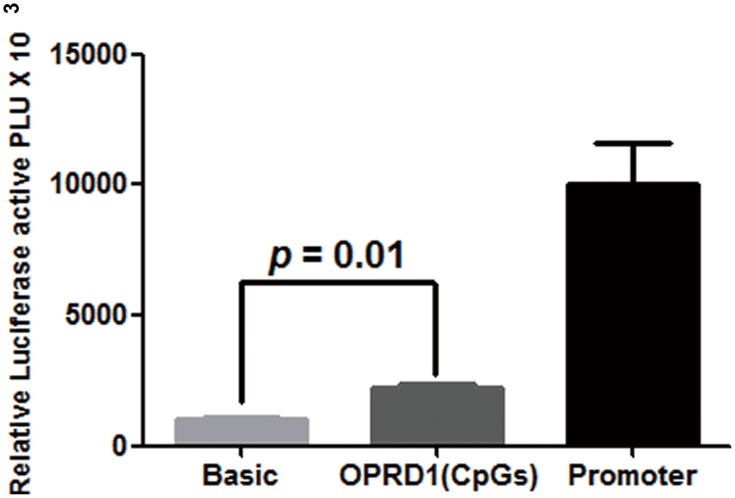
Effects of *OPRD1* promoter region on transcription activity*. *OPRD1 (CpGs) stands for the fragment containing the pyrosequenced sequence. The pGL3-Basic as negative control and pGL3-Promoter vector as positive control are used. pRL-SV40 vector as an internal control receptor is used to normalize the differences in transfection efficiency. Relative luciferase activity is measured in triplicates.

Age is an important factor in AD. Our results show that a female-specific association is found between CpG1 and age in AD patients (r = 0.434, *p* = 0.034, Fig 3). Moreover, as shown in Fig 1, the target region (hg19, chr1:29138224–29138296) in *OPRD1* promoter is overlapped with the binding sites of H3K4me1/3 and mutiple transcription factors. This motivates us to perform luciferase reporter gene assay to explore the regulatory role of the target fragment in gene expression. Recombinant plasmid with insertion of *OPRD1* promoter fragment is shown to have higher luciferase expression than the empty plasmid (Fig 4, fold = 2.2, *p* = 0.01).

## Discussion

AD is newly defined as type 3 diabetes with impairments in the utilization of brain glucose caused by insulin resistance [12]. Brain metabolic dysfunction is the core of AD, and it may eventually cause amyloid-β (Aβ) deposition and neurofibrillary tangles in brain [13]. Opioid and its receptors play important roles in glucose homeostasis [14]. Opioid receptor antagonist is found to be able to dramatically reduce blood glucose in diabetic mice [15] and humans [16]. *OPRD1* encodes delta opioid receptor, which belongs to G-protein-coupled receptor family and plays important roles in homeostasis of potassium [17,18] and glycometabolism [19]. In the current study, *OPRD1* hypermethylation is found to be associated with AD.

*OPRD1* encodes delta opioid receptor which has been found to be significantly decreased in AD brain [20]. A common *OPRD1* variant is shown to be associated with the accumulation of amyloid precursor protein in human neuroblastoma cells [21]. A subsequent cohort study manifests that the *OPRD1* variant is associated with smaller regional brain volumes by increasing the enzyme activities of secretases [22], which may eventually lead to a significantly increased risk of AD. Opioid receptors are distributed in brain and play important roles in multiple brain functions [23–25]. Increased *OPRM1* promoter methylation may lead to gene silencing and worsen the outcomes of neonatal abstinence syndrome [26]. AD is a neurodegenerative disease which is characterized by learning and memory disorder. Mouse *Oprd1* gene expression is shown to be regulated by promoter methylation [27]. In the present research, a significant difference in *OPRD1* CpG3 methylation levels is observed between AD cases and controls in human peripheral blood [27].

The risk of AD is significantly increased along with aging, which effects on DNA methylation modules in human brain and blood [28]. Cerebrospinal fluid (CSF) have shown potential in AD diagnosis with the utilities of Aβ42 and tau components. Similar to the CSF components, the biochemical alternations in peripheral blood have consistent tendency with their levels in the central nervous system. The mononuclear cells in blood have shown correlation with AD pathology through the inflammatory process [29]. The increased expression levels of peptidyl-prolyl cis/trans isomerase (Pin1) in peripheral blood mononuclear cells are consistent with its performance in brain [30]. The age-associated genes are often enriched in potentially functional CpG-methylation sites such as enhancer and insulator regions [31]. Age-associated gene methylation patterns in blood are shown to predict all-cause mortality in later life [32], suggesting that peripheral blood may be a reliable correlate of physiological processes in other tissues [33]. Besides, CpG-island methylation levels are also shown to be generally highly correlated between blood and brain [34]. Moreover, 94% of the methylated mutated CpG sites (CpG-SNPs) in brain are methylated in blood. Concordant methylation alterations are found in brain and blood for biomarker discovery in Parkinson disease [35]. Specifically, sex-dependent effect on *BDNF* promoter methylation levels is found in the various tissues and blood samples [36]. However, recent epigenomics research shows that most DNA methylation markers in peripheral blood do not reliably predict brain DNA methylation status [37]. The tissue specificity of DNA methylation profile may also cause the discrepancy of DNA methylation between blood and brain. Previous principal component analysis have identified the different patterns of DNA methylation in brain and blood tissues [38]. A significant hypomethylation of TNF-α promoter is found in AD patients' brains but not in their blood [39]. However, this doesn’t discount the utility of applying a blood-based epigenetic study to identify biomarkers of brain disease related biochemical parameters [40]. One possible explanation for the discrepancy of DNA methylation between blood and brain is the fact that DNA methylation results are significantly relying on DNA integrity of post mortem interval [41].

In the current study, differently methylated *OPRD1* between AD patients and controls may be likely to provide a biomarker in AD, although it is unable to predict its function in brain. In the previous studies, we have found that hypermethylated *OPRK1* and *BDNF* promoters are associated with the risk of AD [5,6]. Here, we find that hypermethylation of another gene, *OPRD1*, is associated with AD. We further correlate the methylation levels among the three genes, and find there are very few significant correlations (S2 and S3 Figs). This suggests that the contribution of *OPRD1* methylation to AD is independent of the previous two genes.

In the current study, the luciferase reporter assay has shown that the expression level is elevated in the recombinant plasmid with insertion of *OPRD1* promoter fragment compared with empty plasmid. Our study suggests that the target sequence from *OPRD1* is related to gene expression. An online data mining shows that DNA methylation inhibitor 5-aza-2’-deoxycytidine (5AdC) is able to increase *OPRD1* expression (Accession GSE38823, fold change = 1.18, *p* = 0.030, S4 Fig) [42]. Further study on the mechanisms under methylation of target sequence of *OPRD1* promoter is still needed.

Homocysteine in high concentration may deteriorate dementia and serve as a critical regulation function in DNA methylation modification though modulating the activity of methyltransferase [43,44]. Our result is in line with the previous evidence [45,46], although no correlation between homocysteine and *OPRD1* methylation is identified.

Oxidative stress plays an important role in the development of AD [47]. Antioxidants, such as TBIL, have been reported to be decreased in AD patients [48]. In the current study, a significant reduction of these antioxidants is found in the AD group, which is consistent with the previous study [49]. Further study is needed to elaborate the relationships between these antioxidants and methylation.

Alterations in lipoprotein metabolism have been reported to be involved in AD. HDL-C is an essential part of cholesterol in both the peripheral nervous system and the central nervous system [50]. The correlation of HDL-C with CpG3 methylation in female controls provides clues to elaborate the mechanisms by which lipoprotein metabolism contributes to AD. Significant increases in K concentration in the cerebellum are found in AD samples compared with the controls, and a similar trend is observed in murine astrocytes after treatment with Aβ_25–35_ peptide in a previous study [51].

There are several limitations. Firstly, methylation of DNA extracted from the whole blood is contributed by the mixed methylation of DNA from different types of blood nucleated cells [52]. Distribution of different blood cell types may serve as a confounding factor in whole-blood DNA methylation study [53,54]. The current study uses whole-blood DNA, and thus the uncertain constitution of blood cell types may potentially affect our results. Secondly, Psychiatric drugs may influence peripheral blood DNA methylation patterns by changing the cell type composition in blood [55]. Our results suggest that robustness in the association of *OPRD1* CpG3 with AD, although we can exclude a chance that drugs may influence peripheral blood DNA methylation patterns by changing the cell type composition in blood [55]. Therefore, influence of drug application on *OPRD1* methylation in AD peripheral blood is awaited to be addressed in the future. Thirdly, the luciferase reporter assay we have performed in this study only have found the association between *OPRD1* promoter and gene expression. The methylation status of the target sequence in pGL3 plasmid is unknown during the transformation into E.coli DH5α strain and co-transfection into HEK293T cells. But the significantly higher expressions of *OPRD1* are found in the different cell lines after treated by DNA methylation inhibitor 5-aza-2’-deoxycytidine (5AdC) in the previous study [56]. The methylation level of *OPRD1* can directly affect the expression of *OPRD1* in many cell lines. Further study on the mechanism under methylation of target sequence of *OPRD1* promoter is still needed. Fourthly, our sample size is small. This may influence the detection power, especially for the subgroup analysis of *OPRD1* methylation with AD. In addition, due to the limited of samples number, the statistical analysis in the current study is only corrected by smoking.

In conclusion, our study implies that the elevated *OPRD1* CpG3 methylation may contribute to the risk of AD through its effect on the gene expression which may subsequently change TBIL, HDL-C and K levels. However, future work is needed to provide further supportive evidence of our hypothesis.

## Supporting information

S1 Fig *OPRD1* methylation levels between various drug-treated AD cases and controls*. *: AD, M, E, A, and O stand for all the AD cases, memantine-treated, exelon-treated, aricept-treated, and other AD cases).
(TIF)

S2 Fig Association of *OPRD1* CpG1-3 methylation with *BDNF* and *OPRK1* methylation in AD cases. (TIF)

S3 Fig Association of *OPRD1* CpG1-3 methylation with *BDNF* and *OPRK1* methylation in controls. (TIF)

S4 Fig Association of *OPRD1* methylation and gene expression. *. Total RNA is collected from oral cancer cell lines before or after 5AdC treatment. The Benjamini and Hochberg method is used to compare the difference of RNA level between before and after 5AdC treatment.
(TIF)
