# Supplementary figures and images for: Elevated OPRD1 promoter methylation in Alzheimer’s disease patients
Source: PLoS One. 2017 Mar 2;12(3):e0172335. doi: 10.1371/journal.pone.0172335 (PMC5333823; doi:10.1371/journal.pone.0172335)

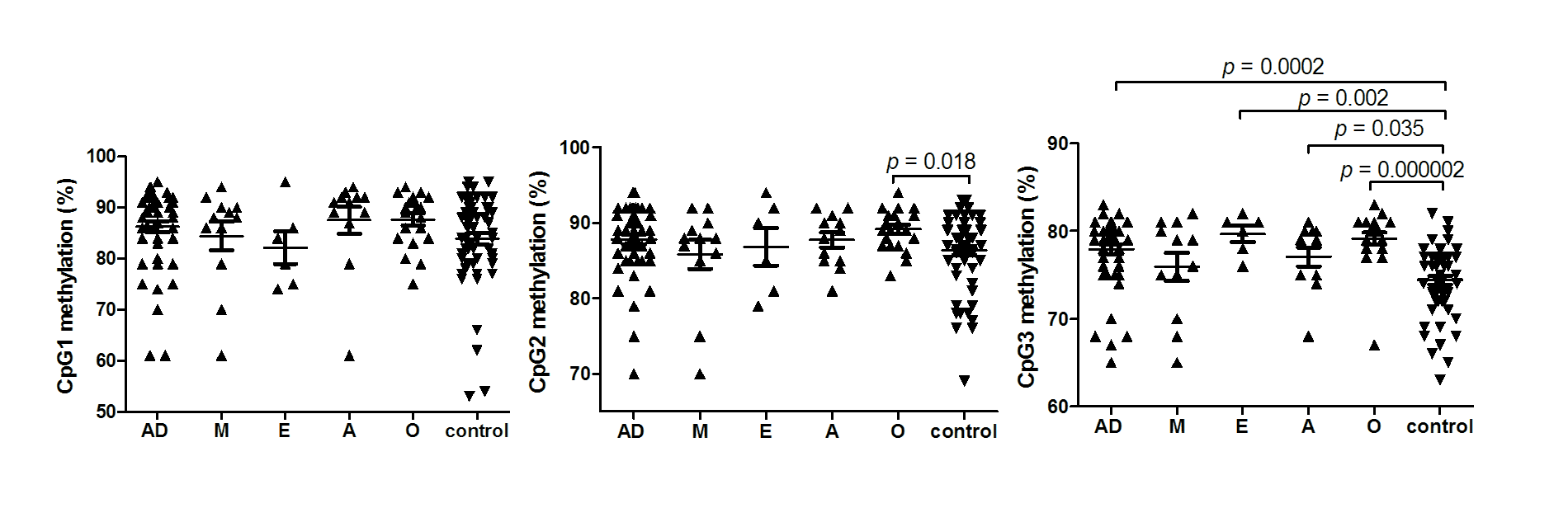

Supplement: S1 Fig — *: AD, M, E, A, and O stand for all the AD cases, memantine-treated, exelon-treated, aricept-treated, and other AD cases). (TIF) [file pone.0172335.s001.tif]

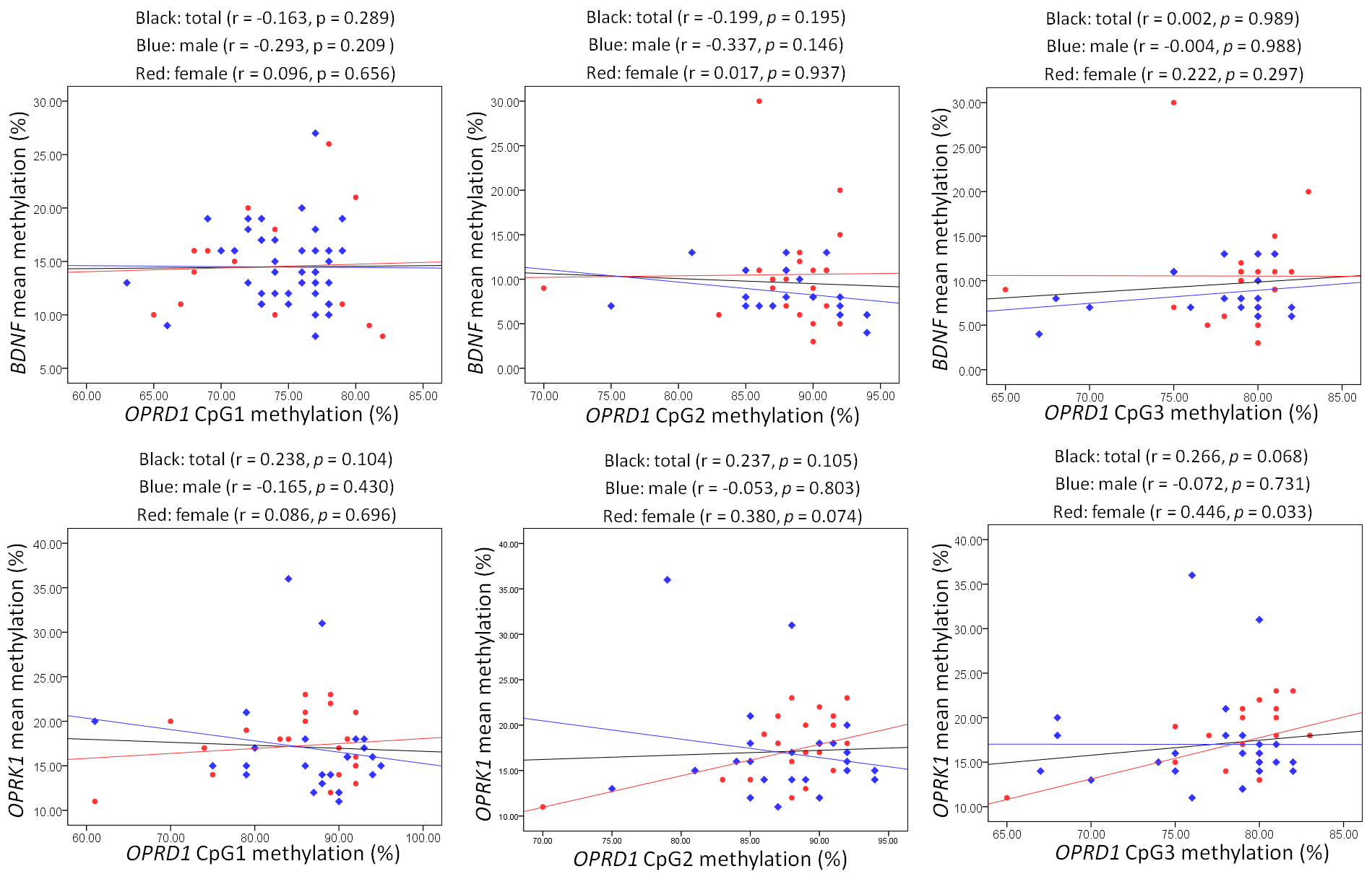

Supplement: S2 Fig — (TIF) [file pone.0172335.s002.tif]

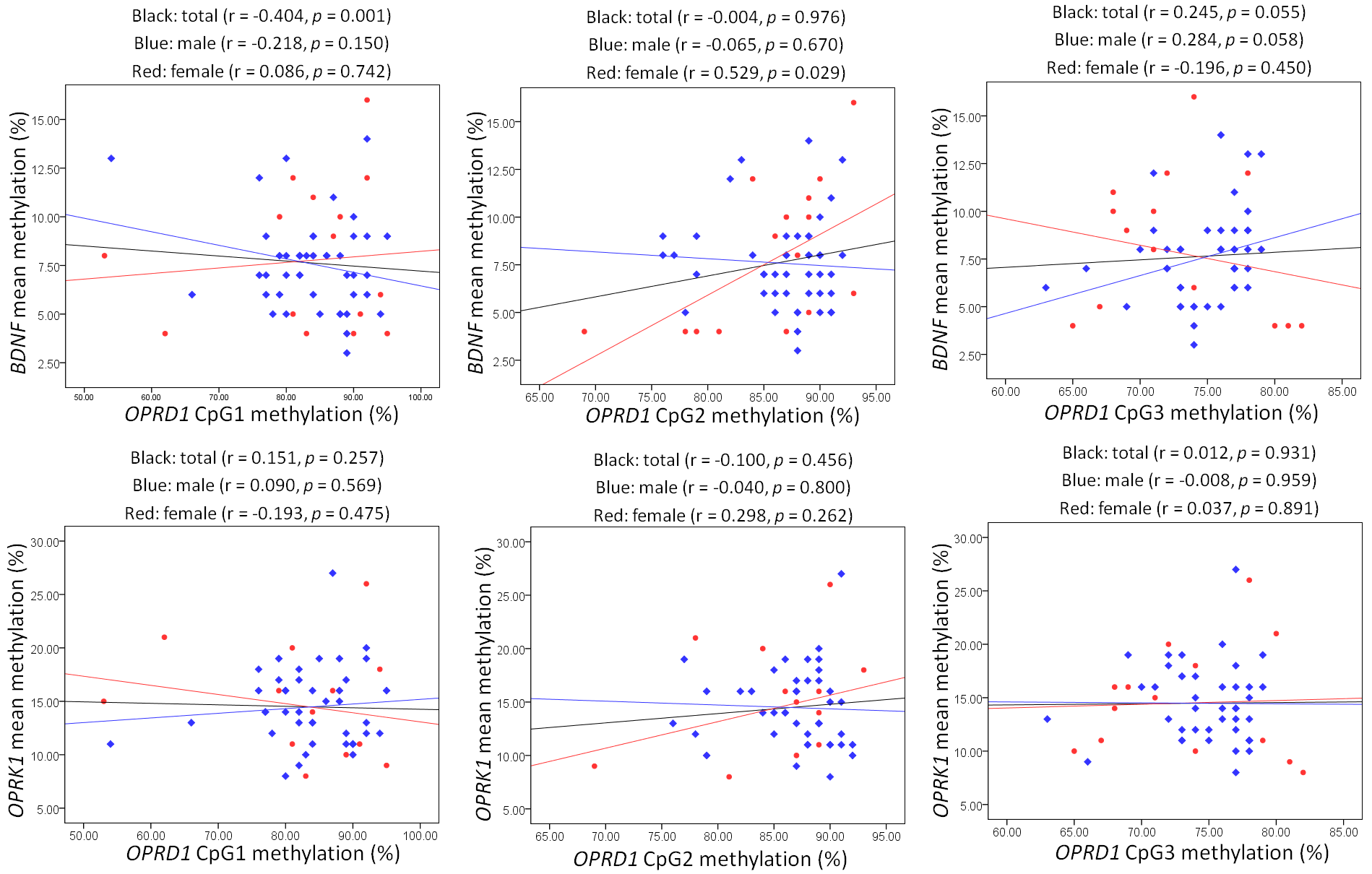

Supplement: S3 Fig — (TIF) [file pone.0172335.s003.tif]
